# DOMe: A deduplication optimization method for the NewSQL database backups

**DOI:** 10.1371/journal.pone.0185189

**Published:** 2017-10-19

**Authors:** Longxiang Wang, Zhengdong Zhu, Xingjun Zhang, Xiaoshe Dong, Yinfeng Wang

**Affiliations:** 1 The School of Electronic and Information Engineering, Xi’an Jiaotong University, Xi’an, Shaanxi, 710049 P.R. China; 2 The Shenzhen Institute of Information Technology, Shenzhen, Guangdong, 518172 P.R. China; Southwest University, CHINA

## Abstract

Reducing duplicated data of database backups is an important application scenario for data deduplication technology. NewSQL is an emerging database system and is now being used more and more widely. NewSQL systems need to improve data reliability by periodically backing up in-memory data, resulting in a lot of duplicated data. The traditional deduplication method is not optimized for the NewSQL server system and cannot take full advantage of hardware resources to optimize deduplication performance. A recent research pointed out that the future NewSQL server will have thousands of CPU cores, large DRAM and huge NVRAM. Therefore, how to utilize these hardware resources to optimize the performance of data deduplication is an important issue. To solve this problem, we propose a deduplication optimization method (DOMe) for NewSQL system backup. To take advantage of the large number of CPU cores in the NewSQL server to optimize deduplication performance, DOMe parallelizes the deduplication method based on the fork-join framework. The fingerprint index, which is the key data structure in the deduplication process, is implemented as pure in-memory hash table, which makes full use of the large DRAM in NewSQL system, eliminating the performance bottleneck problem of fingerprint index existing in traditional deduplication method. The H-store is used as a typical NewSQL database system to implement DOMe method. DOMe is experimentally analyzed by two representative backup data. The experimental results show that: 1) DOMe can reduce the duplicated NewSQL backup data. 2) DOMe significantly improves deduplication performance by parallelizing CDC algorithms. In the case of the theoretical speedup ratio of the server is 20.8, the speedup ratio of DOMe can achieve up to 18; 3) DOMe improved the deduplication throughput by 1.5 times through the pure in-memory index optimization method.

## Introduction

Deduplication is an efficient data reduction technology, and it is used to mitigate the problem of huge data volume in storage systems. At present, deduplication is widely used in storage systems [1, 2], especially in backup systems [3–5].

Database systems are an important part of IT infrastructure and are ubiquitous nowadays. Therefore, how to efficiently carry out deduplication on the database backup data has been an important study. Previous studies have investigated the effect of data deduplication on these data [6, 7].

With the continuous development of semiconductor technology, the database server hardware is about to drastically change. According to HP’s research, future server computers will be equipped with a large number of cores, large Dynamic Random Access Memory (DRAM), and huge non-volatile random-access memory (NVRAM) [8]. As a result, the database architecture will also change. NewSQL databases, such as H-store/VoltDB [9], MemSQL [10] and Hekaton [11], are the focus of the database research community, and may become the mainstream database in the future. As the architecture of NewSQL database and its server hardware drastically changes, it is needed to redesign the deduplication method for NewSQL backup data to optimize the deduplication performance.

The challenge of optimizing deduplication performance is mainly in the following aspects:

First, in recent years, in-memory computing technology has aroused the attention of researchers [12] because of the rising capacity and falling prices of DRAM. Traditional relational database management system (RDBMS) keeps data on disk and maintains buffer cache in memory to improve performance. However, RDBMS thus frequently generates disk I/O, which has been unable to meet the requirements of modern on-line transaction processing system (OLTP). Consequently, the use of in-memory computing technology to improve NewSQL database performance has become a common method. With in-memory computing technology, all data of NewSQL database is maintained in DRAM. Due to the volatile of DRAM, NewSQL data needs to be backed up frequently. In the NewSQL server, the huge capacity NVRAM is suitable for backup. However, the backup between NewSQL and traditional databases have different characteristics. Unlike the data is transferred from a high-performance hard disk to a regular hard disk in traditional database backup, the data is transferred from DRAM to NVRAM in NewSQL. As a result, how to redesign the deduplication method so as not to affect backup performance is a challenge.

Second, an efficient parallel deduplication method is needed. Content defined chunking (CDC) [7] can achieve high duplicate elimination ratio (DER), and therefore is the most widely used data chunking algorithm in deduplication. However, the CDC algorithm needs to repeatedly slide one byte and calculate the Rabin fingerprint value of the backup data stream, which leads to very low performance. The next generation NewSQL server will have thousands of CPU cores. Thus, how to design an efficient parallel deduplication method by utilizing a large number of CPU cores to improve the deduplication performance is a challenge.

To address these challenges, we design a deduplication framework called DOMe to take advantage of next-generation NewSQL server features to optimize performance. DOMe was implemented and evaluated in H-store, which is a typical state-of-the-art NewSQL database. The experimental results show that: 1) DOMe can significantly reduce the duplicated NewSQL backup data. 2) DOMe significantly improves deduplication performance by parallelizing CDC algorithms. In the case of the theoretical speedup ratio of the server system is 24, the speedup ratio of DOMe can achieve up to 18; 3) DOMe improved the deduplication throughput by 1.5 times through the pure in-memory index optimization method.

## Related work

Chunking is the first step in the data deduplication process, in which a file or data stream is divided into small chunks of data so that each can be fingerprinted. The simplest chunking approach is to divide the file/data stream into equal, fixed-sized chunks, an approached referred to as fixed-size chunking (FSC) [5]. In FSC, if a part of a file or data stream, no matter how small, is modified by the operation of insertion or deletion, not only is the data chunk containing the modified part changed but also all subsequent data chunks will change, because the boundaries of all these chunks are shifted. This can cause otherwise identical chunks (before modification) to be completely different, resulting in a significantly reduced duplicate identification ratio of FSC-based data deduplication. To address this boundary-shift problem [13], the content-defined chunking (CDC) algorithm, was proposed in LBFS [14], to chunk files or data streams for duplicate identification. CDC employs Rabin’s fingerprints [15] to choose partition points in the object. Using fingerprints allows CDC to “remember” the relative points at which the object was partitioned in previous versions without maintaining any state information. By picking the same relative points in the object to be chunk boundaries, CDC localizes the new chunks created in every version to regions where changes have been made, keeping all other chunks the same.

Based on CDC algorithm, many researchers proposed improved chunking algorithms [7, 16, 17]. However, most of these methods target at reducing more duplicated data rather than improving performance. To improving performance, the researchers proposed using multi-thread method to speedup deduplication process [18]. But these methods is based on a traditional parallelized programming model and can be further improved to achieve a better performance. Therefore, we propose using fork-join model to speedup deduplication performance. Fork-join is a state-of-art parallelized programming model and can make better use of multi core resources of modern CPU. Although our proposed method is exemplified in CDC, it can also be used for other state-of-art CDC based chunking algorithm.

## Materials and methods

### Framework

**Fig 1:**
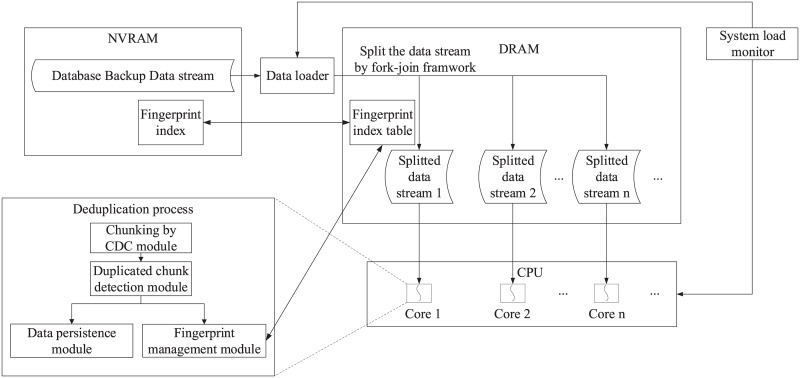
DOMe architecture.

The framework of DOMe is shown in Fig 1. To not affect the performance of NewSQL database, DOMe implements deduplication as post-processing. In this way, NewSQL directly writes the backup data into the NVRAM buffer, then the DOMe carry out the deduplication process when the system is idle. The data loader is used to transfer backup data stream from NVRAM into DRAM for deduplication processing. After loading into the DRAM, the data stream is carried out a parallelized deduplication process. When the task is less than a predefined threshold, the current subtask is assigned to a CPU core and the deduplication process is performed.

### When data deduplication occurs

Deduplication may occur “in-line”, as data is flowing, or “post-process” after it has been written. The advantage of in-line deduplication over post-process deduplication is that it requires less storage, since duplicate data is never stored. On the negative side, because hash calculations and lookups take so long, data ingestion can be slower, thereby reducing the backup throughput of the device. As the NewSQL database requires high backup performance, so we choose post-processing deduplication. Backup data firstly write into NVRAM, when the NewSQL system is relatively idle, and then trigger the de-duplication operation.

### System load monitoring method

Deduplication process requires a lot of CPU resources. To not affect the normal NewSQL transaction processing, the system needs to be more idle when the deduplication is processing, so as not to seize the transaction processing required CPU resources. In the Linux system, we can use uptime command to monitor the system load. When monitoring the system load value within 1 minute is always lower than a pre-set threshold, the system is determined to be idle at this time. The DOMe then reads the backup data to process it.

### Data chunking

Data chunking, the central part of deduplication, divides the data stream into small chunks, which are used as the basic unit for duplicated data detection. DOMe uses content defined chunking (CDC) instead of fixed-sized partition (FSP). FSP divides the data stream into fixed-length chunks of data, requiring only a small amount of CPU computation. Thus has the advantage of high performance, but there is a problem of data update sensitivity. The problem is that after inserting or deleting in the data stream, all the data after the change point will slide forward or backward. And these data will be wrongly detected as new. This situation is avoided with CDC, but because CDC requires to slide the detection window one byte each time and calculates the Rabin fingerprints of the current window, resulting in a large amount of CPU computational. Therefore, its performance is significantly lower than FSP. To address this problem, we proposed a fork-join based parallelization deduplication method to improve the performance.

### Fork-join based parallelization deduplication method

**Fig 2:**
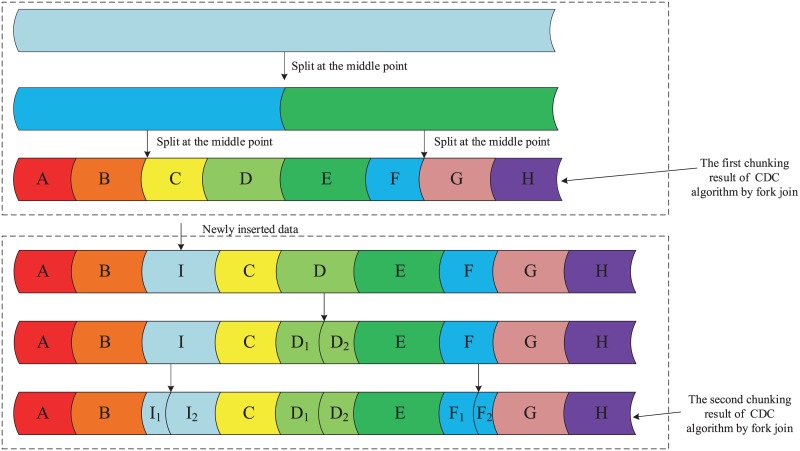
The problem of dividing the data stream at the middle point.

How to effectively parallelize the deduplication process is a complex issue. The use of fork-join framework can significantly simplify the complexity of parallelization deduplication. The fork–join model is a way of setting up and executing parallel programs, such that execution branches off in parallel at designated points in the program, to “join” (merge) at a subsequent point and resume sequential execution. Parallel sections may fork recursively until a certain task granularity is reached. The difficulty of parallelization deduplication with fork-join is how to split tasks. If the task is divided at the midpoint of the data stream, some data chunks are erroneously divided into smaller chunks, which will be identified as new data chunks, resulting in a reduction of the duplicate elimination ratio, as shown in Fig 2.

**Fig 3:**
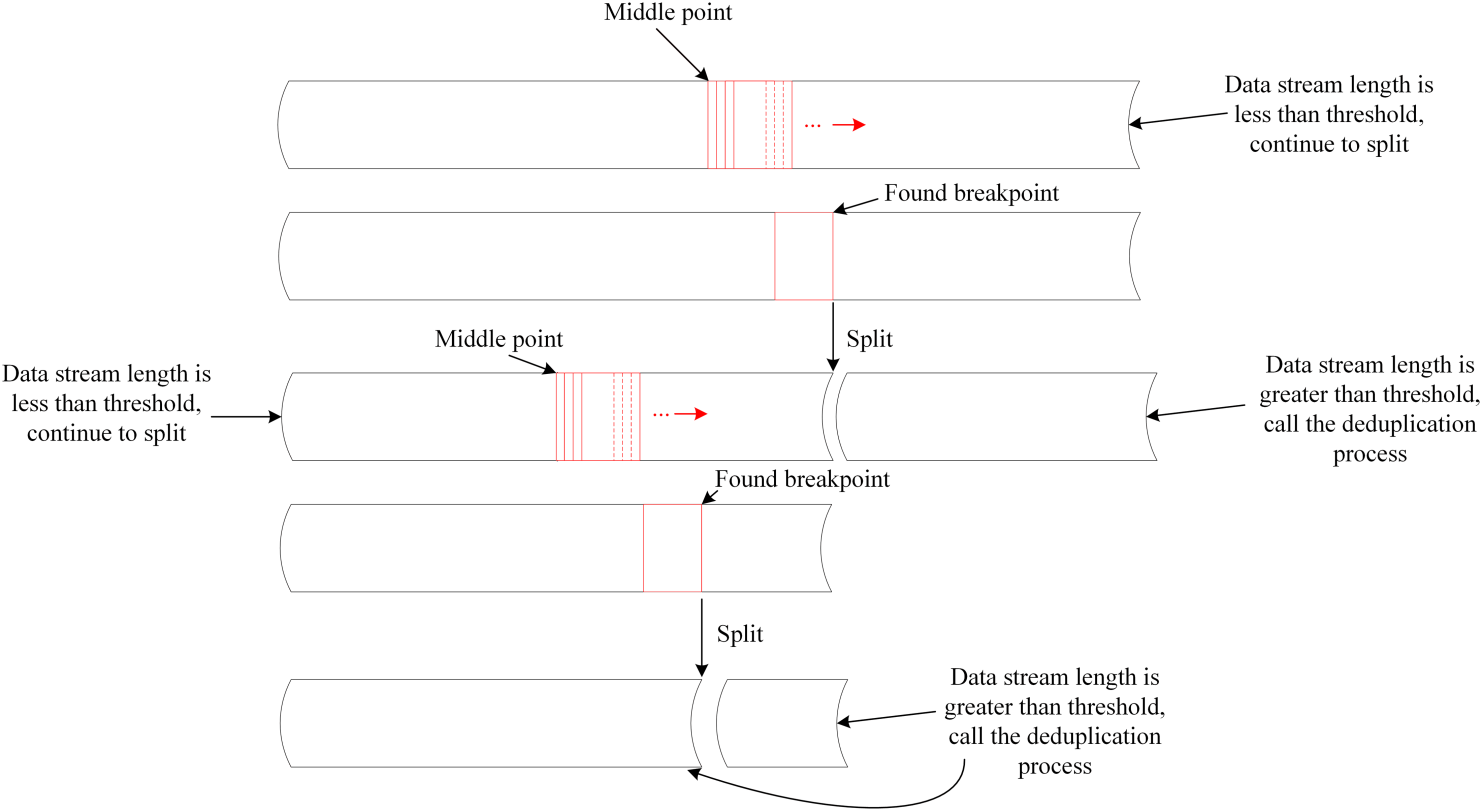
The principal of parallelized CDC algorithm based on fork-join framework.

To solve this problem, we make the division point of fork-join method exactly at the CDC chunking boundary point, as shown in Fig 3. The method is detailed in algorithm 1.

**Algorithm 1** Fork-join based parallelization deduplication algorithm

**Input:** Data stream *ds*

**Output:** None

1: set the sliding window *w*

2: set *w* start at the middle point of the data stream *ds*

3: slide *w* until find a boundary point by CDC algorithm

4: <*ds1*, *ds2*>← split data stream at the end point of the window

5: **if** the length of *ds1* or *ds2* is less than the threshold *T* **then**

6:  do deduplication process

7: **else**

8:  split the *ds1* or *ds2* by this algorithm recursively

### Duplicated data detection

The purpose of duplicated data detection is to identify the unique data among current and all stored backup data. The duplicated data detection is based on the following properties of MD5 (or SHA-1, and so on) function.

*Property 1* [19]: For two different data chunks, the probability of their corresponding MD5 hash value collision (i.e., the same) is 2^−128^. Each unique data chunk has a unique hash value because probability is very low in practice.

Based on the above property, the MD5 hash value can be referred to the image of a fingerprint. Therefore, the duplicated data detection of each chunk can be achieved by checking the fingerprint, which can be formally defined as follows:

*Definition 1* Duplicated data detection. Set *A* is defined as *A* = {*c*_*i*_|*c*_*i*_ is the data chunk already written}. Set *B* is defined as *B* = *h*_*i*_|*h*_*i*_ = hash (*c*_*i*_). For a new chunk *c*, the duplicated data detection calculates fingerprint *h* = MD5 (*c*) and determines whether *h* ∈ *B* is established. If true, the data chunk *c* is considered duplicated; otherwise, it is considered a unique chunk.

**Algorithm 2** Duplicated data detection

**Input:** Data stored in *circBuf*

**Output:** Chunking result

1: *fpTable*←initHashTable 〈 String, long 〉 ()

2: *fpTable*←initHashTable 〈 String, long 〉 ()

3: *metafile*←fopen(backup stream)

4: **while** *circBuf* is not empty **do**

5:  *chunk* ← get(*circBuf*)

6:  *fp*← MD5(*chunk*);

7:  *isDedup*←isHashExist(*fp*, *fpTable*)

8:  **if** not *isDedup* **then**

9:     *offset*← append chunk to the end of current chunk store file

10:   write the 〈*fp*, (*chunkstoreFileName*, *offset*)〉 to fingerprint index

11:  **else**

12:   (*chunkstoreFileName*, *offset*)←get(*fp*, *fpTable*)

13:  fwrite(*metafile*, *chunkstoreFileName*, *offset*)

Data detection process can be detailed in algorithm 2. When the DOMe is started, a purely in-memory hash table is initialized as the fingerprint index (line 1). In a backup, the *ID* (the database name + backup timestamp) is used as the file name to create a metadata file (line 2). Then, the buffer is constantly checked to find any chunk exist (line 3). When a data chunk is read (line 4), its MD5 value is calculated as the fingerprint *fp* (line 5). The fingerprint index is searched to find whether *fp* exists (line 6). If *fp* is not found (line 7), the current chunk can be considered unique. The unique chunk is appended to the chunk store file. Afterward, the offset of the start position of the chunk is returned (line 8). Furthermore, the tuple 〈fp, (chunkstoreFileName, offset)〉 is inserted to fingerprint index (line 9). If the *fp* is found in fingerprint index (line 10), the current chunk is duplicated and the chunk need not be written. The chunk information (chunkstoreFileName, offset) can be obtained from the fingerprint index according to the *fp* (line 11). Finally, regardless of whether the current block is duplicated, the chunk information (chunkstoreFileName, offset) needs to be written to the metadata file of the current backup for data recovery (line 13).

### Fingerprint index management

Fingerprint index holds all corresponding fingerprint and location information of all written chunks. Due to the limited memory capacity of the server, the traditional method saves the fingerprint index table on disk and maintains the cache in memory. However, the server system of NewSQL has a large amount of memory. To optimize deduplication performance, DOMe maintains fingerprint index in memory and only periodically written to disk while the NewSQL database system is idle.

### Data persistence

Three kinds of data: unique data, metadata and fingerprint index, need to be persisted in DOMe. 1) Unique data. The data that was detected as unique need to be written into chunk store file on the file system. 2) Metadata. For every backup, a corresponding metadata file is established, each line in which consists of a chunk information *m*, which is defined as *m* = <*n*, *o*, *s*>. *n* represents the chunk store file name; *o* represents the offset of the chunk in chunk store file; *s* is the size of the chunk. Regardless of whether the chunk is duplicated, the corresponding information *m* of the chunk must be written to the metadata file for database recovery. 3) Fingerprint index. As described above, fingerprint index is maintained purely in memory and only periodically written to disk while the database system is idle. Fingerprint index may lose the data that is written after last persistence if a failure occurs, but it only cause a misjudgment of some duplicated data that will be written in future, leading to small waste of storage space. Therefore, it is not a serious problem.

## Implementation

**Fig 4:**
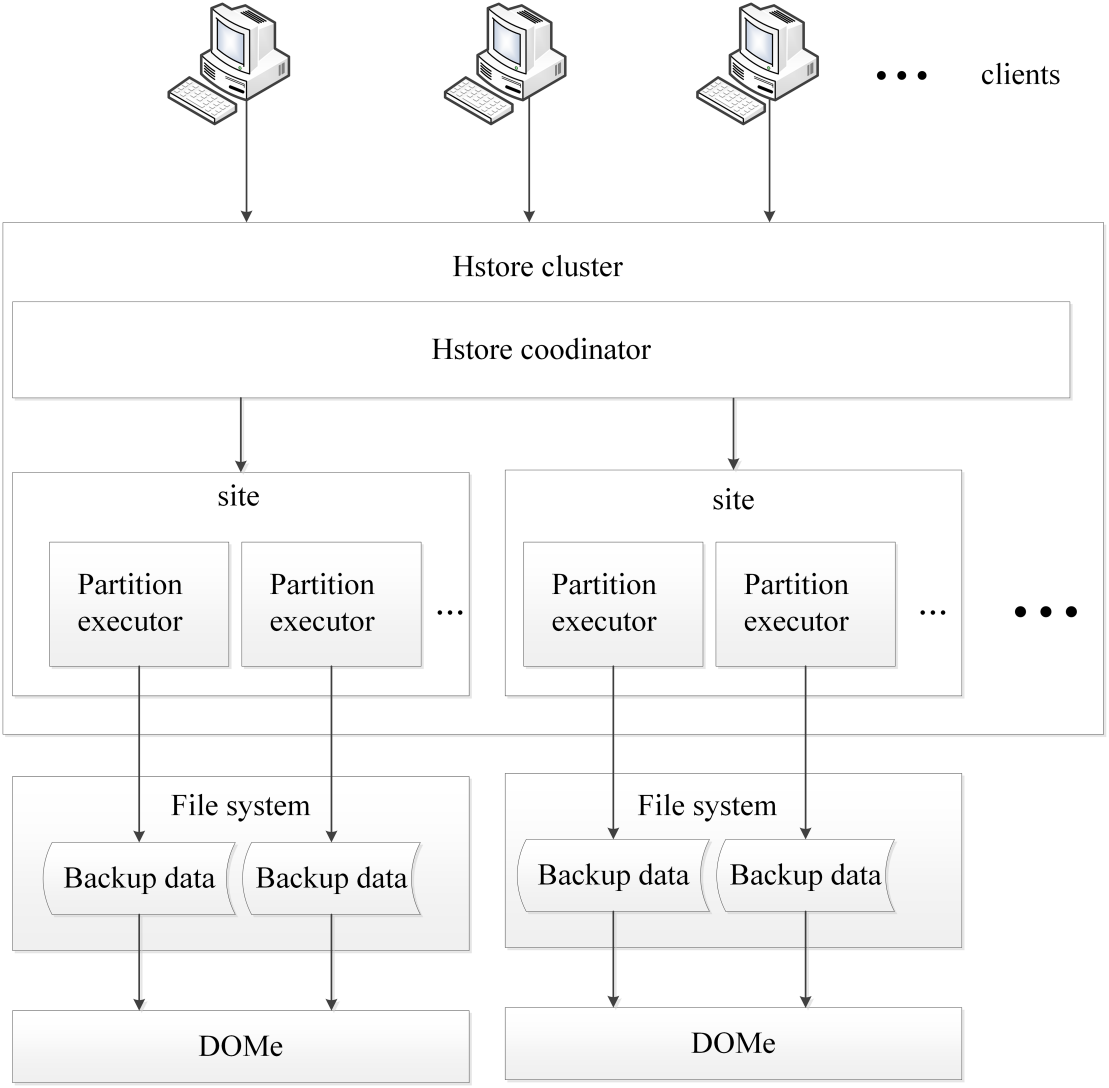
Implementation of DOMe in H-store.

This chapter chooses H-tore [20] as a typical NewSQL test platform. H-store is a next generation of database management system developed by Brown University, Massachusetts Institute of Technology, and Yale University for on-line transaction processing applications. H-tore can provide high throughput, low latency SQL operation, H-store takes full advantage of large memory capacity features of modern server systems. The main code of H-store is implemented in Java, but its SQL execution engine is implemented in C ++. H-store has been commercialized and its commercial version is VoltDB. VoltDB has a very excellent transaction performance, and claims its performance is 100 times higher than traditional database system. H-store represents the future development trend of the database technology. The DOMe implementation architecture is shown in Fig 4. H-store is a typical NewSQL database architecture, using non-page data management. An H-store cluster consists of a number of shared-nothing nodes, each of which contains several partition execution engines as the actual execution unit. Each backup execution engine serializes the respective tuple data when performing a backup and write to the hard disk. Since the data is horizontally distributed across different partition execution engines, the backup data only has significant duplicate data in the same partition, so the DOME method is implemented in each individual partition execution engine.

## Results

### Experimental setup

All the experiments in this section are run on a single server, the configuration of which is below:

1 CPU: 2 Intel Xeon E5-2650 v2 2.6GHz 8-core processors, provide 32 hardware threads by hyper threading technology;
2 RAM: 128GB DDR3;
3 NVRAM: As we can not obtain a NVRAM device currently, we use a 600G MLC PCIe SSD to simulate NVRAM. We compared the key features of our SSD with the Intel^®^ Optane^™^ [21] below, which uses the 3DXPoint technology and is the only available NVRAM device currently.

As shown in Table 1, our SSD has a same interface and similar performance with the Intel^®^ Optane^™^. Therefore, we use this SSD to simulate the NVRAM is reasonable.

**Table 1:** Comparison of our SSD and Intel Optane.

Device	Interface	Random read(4KB) IOPS	Random write(4KB) IOPS
Our SSD [22]	PCIe 3.0 x 4	615K	130K
Intel^®^ Optane™ [21]	PCIe 3.0 x 4	550K	500K

4 Operating system: CentOS release 6.5 (Final);
5 File system: EXT4.

We implemented DOMe method in Java, and use two benchmarks run over H-store to evaluate the DOMe performance. To verify the effectiveness of DOMe, we compared this method with MUCH [18], which is based on a traditional parallel programming model.

### Benchmark

#### TPC-C

Benchmark 1 is the TPC-C. In TPC-C benchmark specification [23], a complex and representative OLTP application is simulated, which assumes a large commodity wholesaler exists. Several commodity library are distributed in different regions. Each warehouse is responsible for supplying ten sales points, and each sales point is responsible for providing service to 3000 customers. Each order of a customer has ten items, and approximately 1% of all orders are out of stock in its own direct warehouse. Thus, other regional warehouses are needed for supply. The main transaction causing the database size increment is Neworder. Parameter configuration is as follows: the warehouse number is set to 1; the running time is set to 1 hour; the automatic backup interval is set to 5 minutes.

#### Stock trading

**Fig 5:**
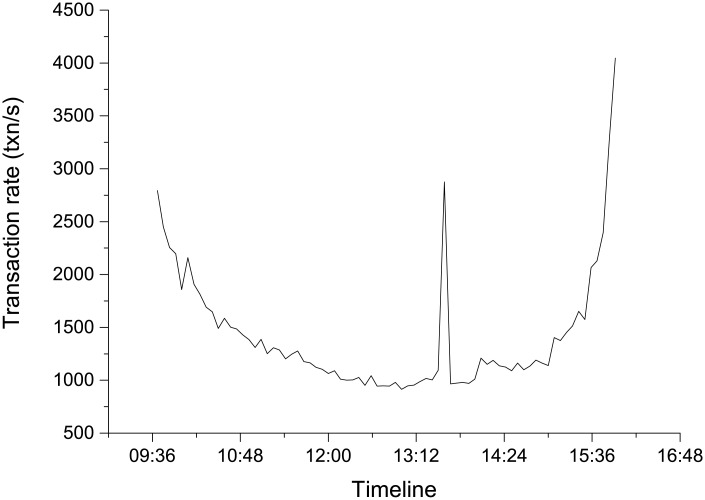
Average transaction rate of S&P 500 from 2015-3-11 to 2015-4-24.

Benchmark 2 simulates real stock trading activities, the database structure in which comes from real stock trading systems. In this system, stock trading will causing the insert of the order information table STK ORDER and trading records table STK MATCHING. Therefore, the benchmark transactions are implemented as the two tables insert operations. To simulate the real stock transaction rate, the S&P 500 [24] transaction rate information is used, which can reflect the key information of the US stock market. The interval of the S&P 500 transaction rate is 5 minutes. The data date range is from 2015-3-11 to 2015-4-24, and an average value among these days’ data is used. Because the S&P 500 trading rate is too high for the experimental server to achieve, in the experiment, the transaction rate has been set to 1/10 of the S&P 500 original rate, as shown in Fig 5. Parameter configuration is as follows: the running time is set to 6.5 hours; the automatic backup interval is set to 30 minutes.

### Data size

In order to verify the validity of the parallelized CDC algorithm in the DOMe method, we compare the data size processed by parallelized and single-threaded CDC algorithm.

**Fig 6:**
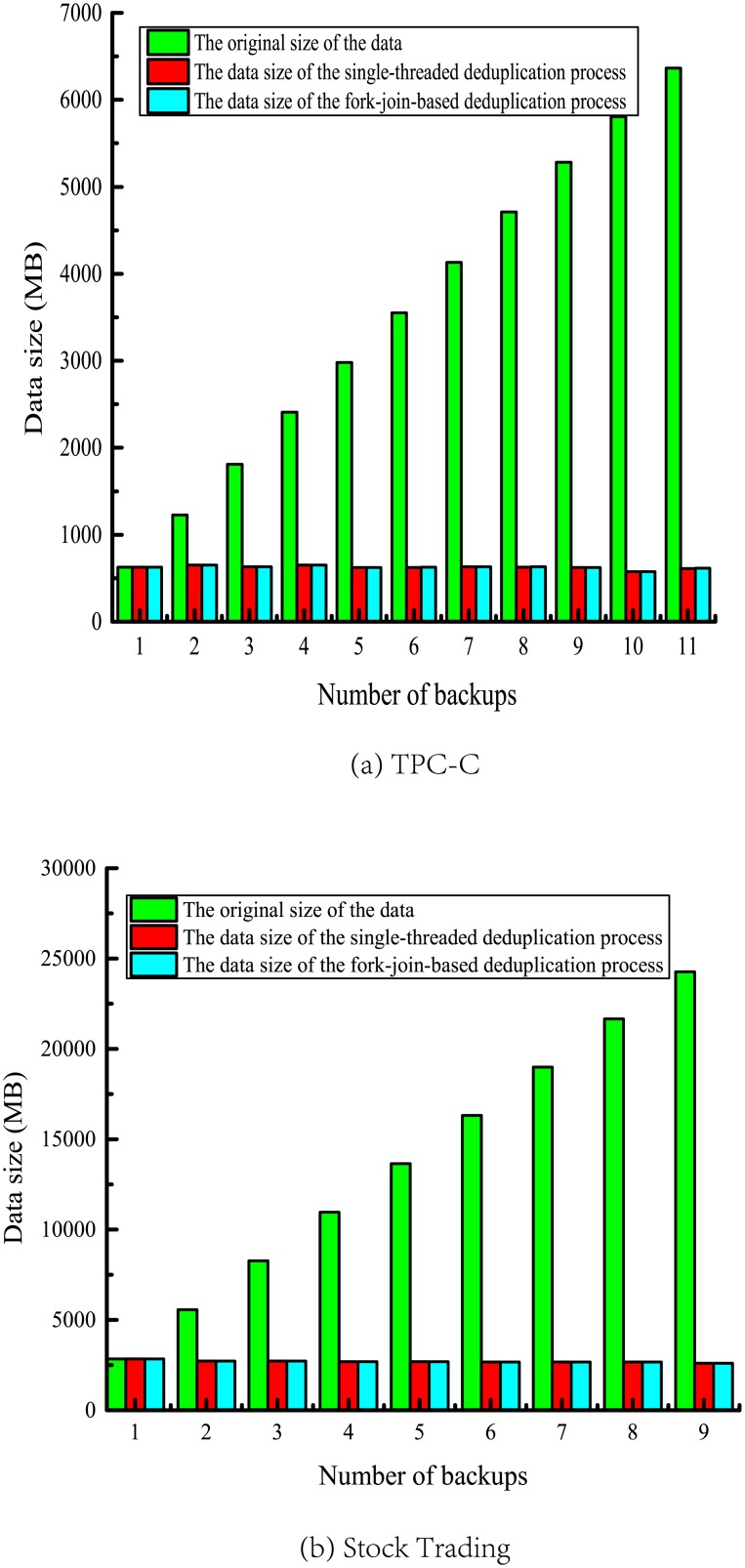
Data size comparison.

Fig 6 is the test result of running DOMe with two benchmark backup data. The results show that the data size is significantly reduced after deduplication process, and also show that the data size of the parallelized CDC algorithm is the same as the single-threaded one, validating the effectiveness of parallelized CDC algorithm.

### Performance of parallelized CDC algorithm

This section compares the performance of DOMe with the deduplication throughput *T*, which is defined in Eq 1.

$$\begin{array}{c} T=\frac{S}{t} \end{array}$$ (1)

**Fig 7:**
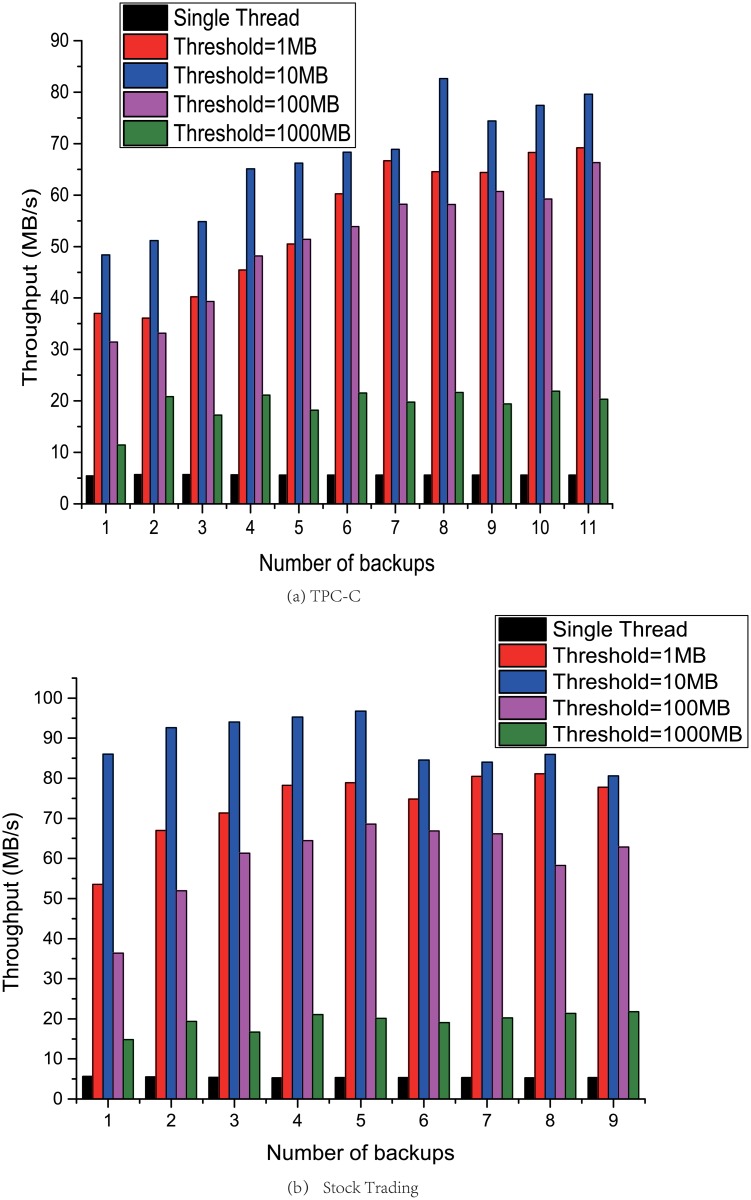
Performance comparison between single thread deduplication and DOMe with variable threshold.

Where *S* represents the processed data size, *t* represents the spent time. Fig 7 are the test results of parallelized and single-threaded CDC algorithm deduplication by TPC-C and stock trading benchmark. To find the best threshold to optimize the throughput of DOMe. We compared several performance result under different threshold. The results show that: a) First, the performance of the parallelized CDC algorithm based on fork-join in DOMe is significantly higher than that of single-threaded CDC algorithm. We use the speedup ratio to reflect the performance improvement effect, according to the definition of speedup and throughput, we define the speedup ratio *S* in Eq 2:

$$\begin{array}{c} S=\frac{T_{m}}{T_{s}} \end{array}$$ (2)

Where *T*_*m*_ is the deduplication throughput for multiple threads, and *T*_*s*_ is the deduplication throughput for single threads.

According to Amdahl’s Law, if we consider that there is no part of the task can not be parallelized, the theoretical maximum speedup ratio should equal to the number of CPU cores. Since the test server CPU has 16 cores, with the Hyper-Threading function, which can achieve up to 30% performance improvement claimed by Intel [25], the theoretical maximum speedup ratio is 20.8(16*130%). The results show that the highest speedup ratios in the two test results are up to 16 and 18, respectively. Therefore, the parallel algorithm proposed by the DOMe method can significantly improve the deduplication throughput. B) The results show that setting the threshold too large or small will degrade performance. When the setting is too large, DOMe can not take full advantage of CPU parallelization. Whereas, setting too small will cause frequent scheduling of threads. The experimental results show that setting threshold to 10MB is reasonable.

**Fig 8:**
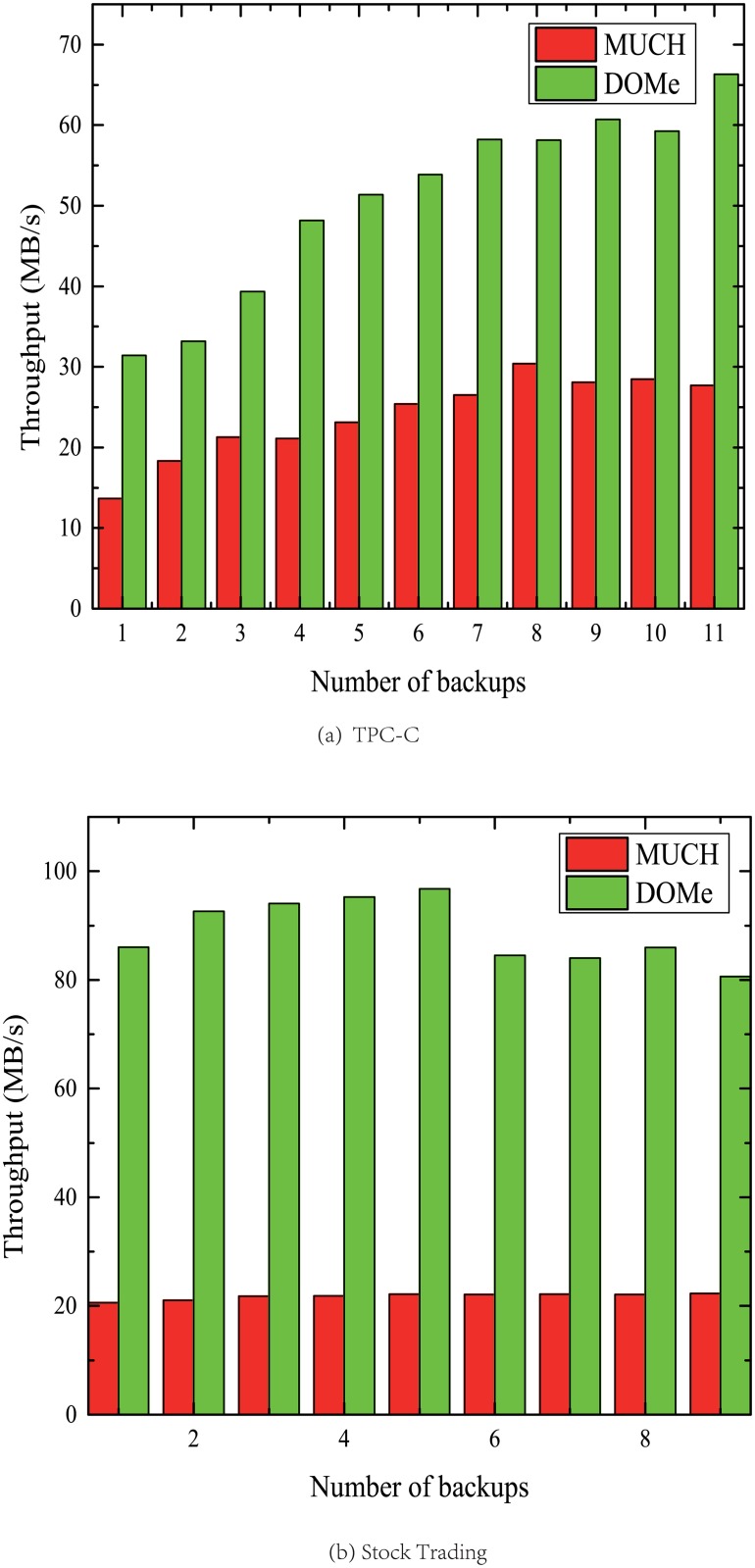
Performance comparison between MUCH and DOMe.

We also compared the throughput of deduplication with MUCH [18], which is a traditional and widely used parallelized deduplication method. The basic idea of MUCH is straight forward. It partitions a file into small fractions called segments and distributes them to the chunker threads. The chunker thread partitions the allocated fraction of the file into chunks. For fair comparison, both the threshold of DOMe and the segments size of MUCH are set to 10MB. As shown in Fig 8, the throughput of DOMe method outperforms MUCH. This is because the fork-join framework uses a work-stealing algorithm. Worker threads that run out of things to do can steal tasks from other threads that are still busy. Therefore, the fork-join based method DOMe can make better use of CPU resources and achieve higher performance.

### In-memory fingerprint index performance

In this section, to evaluate the performance improvement of holding fingerprint index in-memory, we compared the performance of holding index table in memory and in disk with two benchmarks. We still use the throughput *T*, which is defined in Eq 1, to evaluate the performance.

**Fig 9:**
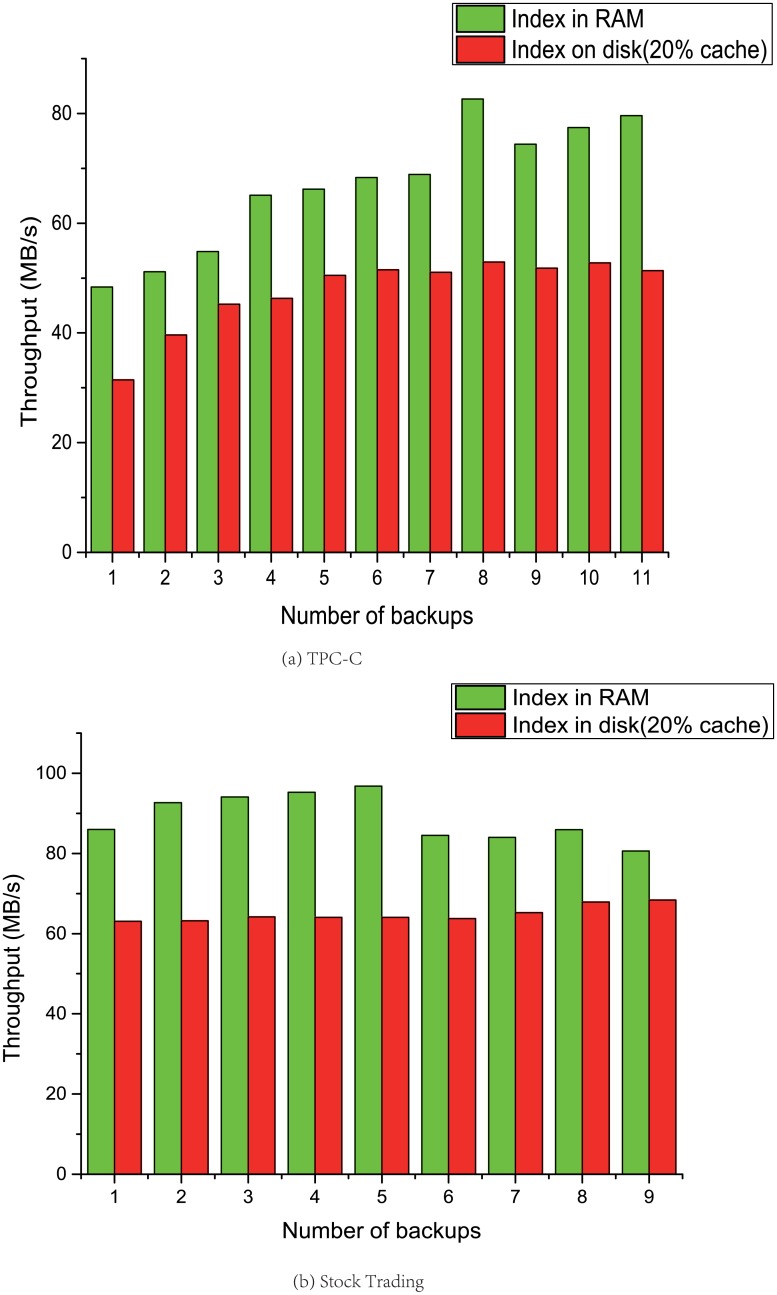
Performance comparison between index in RAM and on disk.

The test results are shown in Fig 9. In the two test results, the deduplication throughput of holding fingerprint index in-memory is 1.5 times higher than holding index in disk and maintains cache in memory. This is because the traditional method of holding index in disk generates too many I/O operations. And even using SSD will still significantly reduce the performance. This is because the performance of disk is still several orders of magnitude worse than memory, and this partially missed fingerprint query operation significantly reduces overall deduplication throughput. The DOMe method eliminates the I / O operations in the index operation by using the pure in-memory index, which significantly improves the deduplication throughput by utilizing the large capacity memory of NewSQL system.

## Conclusions

In this paper, a deduplication-based framework called DOMe was proposed for NewSQL database backups. DOMe could effectively reduce backup data size by deduplication. H-store was used as the experiment platform to evaluate DOMe. The experimental results show that: 1) DOMe can reduce the duplicated NewSQL backup data. 2) DOMe significantly improves deduplication performance by parallelizing CDC algorithms. In the case of the theoretical speedup ratio of the experimental server is 24, the speedup ratio of DOMe achieve up to 18; 3) DOMe improved the deduplication throughput by 1.5 times through the pure in-memory index optimization method. All the experimental data can be obtained in S1 and S2 Tables.

## Supporting information

S1 Table The experimental result of TPC-C. (CSV)

S2 Table The experimental result of stock trading. (CSV)
